# Biological Guided Carbon-Ion Microporous Radiation to Tumor Hypoxia Area Triggers Robust Abscopal Effects as Open Field Radiation

**DOI:** 10.3389/fonc.2020.597702

**Published:** 2020-11-19

**Authors:** Qingting Huang, Yun Sun, Weiwei Wang, Lien-Chun Lin, Yangle Huang, Jing Yang, Xiaodong Wu, Lin Kong, Jiade Jay Lu

**Affiliations:** ^1^ Department of Radiation Oncology, Shanghai Proton and Heavy Ion Center, Shanghai, China; ^2^ Department of Radiation Oncology, Shanghai Proton and Heavy Ion Center, Fudan University Cancer Hospital, Shanghai, China; ^3^ Shanghai Engineering Research Center of Proton and Heavy Ion Radiation Therapy, Shanghai, China; ^4^ Department of Research and Development, Shanghai Proton and Heavy Ion Center, Shanghai, China; ^5^ Department of Medical Physics, Shanghai Proton and Heavy Ion Center, Shanghai, China

**Keywords:** carbon ion, microporous radiation, hypoxia, ^18^F-FMISO PET/computerized tomography, abscopal effect

## Abstract

Recently, a growing number of studies focus on partial tumor irradiation to induce the stronger non-target effects. However, the value of partial volume carbon ion radiotherapy (CIRT) targeting hypoxic region of a tumor under imaging guidance as well as its effect of inducing radiation induced abscopal effects (RIAEs) have not been well investigated. Herein, we developed a technique of carbon ion microporous radiation (CI-MPR), guided by ^18^F-FMISO PET/computerized tomography (CT), for partial volume radiation targeting the hypoxia area of a tumor and investigated its capability of inducing abscopal effects. Tumor-bearing mice were inoculated subcutaneously with breast cancer 4T1 cells into the flanks of both hind legs of mouse. Mice were assigned to three groups: group I: control group with no treatment; group II: carbon ion open field radiation (CI-OFR group) targeting the entire tumor; group III: partial volume carbon ion microporous radiation (CI-MPR group) targeting the hypoxia region. The tumors on the left hind legs of mice were irradiated with single fraction of 20 Gy of CIRT. Mice treated with CI-MPR or CI-OFR showed that significant growth delay on both the irradiated and unirradiated of tumor as compared to the control groups. Tumor regression of left tumor irradiated with CI-OFR was more prominent as compared to the tumor treated with CI-MPR, while the regression of the unirradiated tumor in both CI-MPR and CI-OFR group was similar. Biological-guided CIRT using the newly developed microporous technique targeting tumor hypoxia region could induce robust abscopal effects similar to CIRT covering the entire tumor.

## Introduction

The therapeutic effects of ionizing radiation are often limited by the hypoxia of tumors (1–4). Hypoxia plays important role in radiation resistance, angiogenesis, and metastatic potential (5). Various strategies include the combined use of chemotherapy or agents that directly target the hypoxic cells to increase the radiosensitivity with radiotherapy. However, local failure due to insufficient response to combined treatment modality, remains a major mode of treatment failure (6–9).

Recently, the results of a growing number of *in vitro* and *in vivo* studies indicate that, in addition to the local therapeutic effects, radiation therapy may be in favor of changing the tumor microenvironments correlated with immunity and endothelial cells of the tumor micro-vasculatures, thereby inducing the non-target effects, such as radiation induced bystander effects and abscopal effects (RIAEs) (10–13). RIAEs are radiation induced effects in unirradiated tumors distant from irradiated target regresses. Both pre-clinical and clinical studies have confirmed the existence of abscopal effects and its potential antitumor effects (14–16). Furthermore, Tubin *et al*. demonstrated that the exposure of lung cancer cells to hypoxia and irradiation of hypoxic cells triggered significant RIAEs (17). Additionally, the hypothesis of targeting the hypoxic area of the tumor with a high dose photon-based radiation in attempts to induce an intentional RIAEs in oligometastatic patients was tested (18–20), indicating that the hypoxic area in tumor may be a critical factor contributing to RIAEs from radiation. Massaccesi *et al*. also adopted the technique of high single-dose partial irradiation targeting the hypoxic tumor segment for patients with recurrent bulky lesions, and demonstrated anti-tumor efficacy in their initial clinical results (21). However, the mechanisms through which irradiation exerts its immune modulation effects, are not well clarified.

Based on these preliminary investigations, a growing number of studies focus on partial tumor irradiation with high-dose hypofractionation or single high dose schedule, with an aim to potentially increase the therapeutic efficacy for bulky tumors (14, 15). Of all types of ionizing radiation beams, carbon ion beams are featured with Bragg peak as it has higher linear energy transfer (LET) and relative biological effectiveness (RBE), compared to those of photon and proton (22). Ionization radiation beams of higher LET have been shown to induce more complex DNA damage, despite reportedly more effective in eradicating tumor cells under hypoxic environment, as compared to those with lower LET (23–27). Theoretically, the physical and biological advantages of carbon ion radiation therapy (CIRT) make it more appropriate in the management of bulky or radio-resistant tumors (28–30). Results of pre-clinical or retrospective studies have confirmed its advantages in tumor proliferation and metastasis over photon (31–33). Nevertheless, the value of partial volume CIRT targeting hypoxic region(s) of a tumor under imaging guidance, as well as its effect of inducing RIAEs, have not been well investigated.

^18^F-Fluoromisonidazole (^18^F-FMISO) as hypoxia PET imaging probe has commonly been applied for hypoxic imaging in clinic, and will occur redox reactions under the action of xanthine oxidase and stably combine with some cellular components, thereby reflecting the degree of hypoxia in solid tumors (34, 35). Herein, we developed a type of microporous radiation technique of CIRT (CI-MPR), guided by ^18^F-FMISO PET/computerized tomography (CT), for partial volume radiation targeting the hypoxia area of a tumor and investigated its capability of inducing abscopal effects. This study provides the basis for further investigations for exploring the underlying mechanisms of immune modulating effect induced by CIRT.

## Material and Methods

### Cell Line

The mammary carcinoma cell line 4T1 (closely mimic stage IV human breast cancer) was obtained from the American Type Culture Collection (ATCC). The cells were cultured in Dulbecco modified Eagle medium (DMEM) with 10% fetal bovine serum (FBS) at 37°C in a humidified atmosphere of 5% CO2.

### Animal Experiments

All animal experimental protocols and procedures were approved by the ethics committee of the SPHIC. Four-to-five-week-old and female BALB/c mice were purchased from Shanghai SLAC Laboratory Animal Company and required to acclimate for a week before experiment. The mice were maintained in the specific pathogen free (SPF) environment.

For experiments in which tumor-bearing mice were used, mice were inoculated subcutaneously with 1*10^6^ 4T1 cells into the flanks of both hind legs of mouse for initiating tumor. Tumors were allowed to grow to an area of 7*7 mm before irradiation and systematized within 10% differences in the intra-and inter-tumor volumes. Tumor volume was calculated with the following formula: L × W^2^ × 0.52 (L was the longest diameter and W was orthogonal to L). The volume was measured every other day after radiation until tumor size reached 10% of the mouse’s body weight. Tumor volume at each time point (Vt) was normalized to the initial volume (V0), and the fold change in tumor volume was calculated.

### ^18^F-Fluoromisonidazole Micro-PET/Computerized Tomography Imaging and Quantitative Analysis

**Figure 1:**
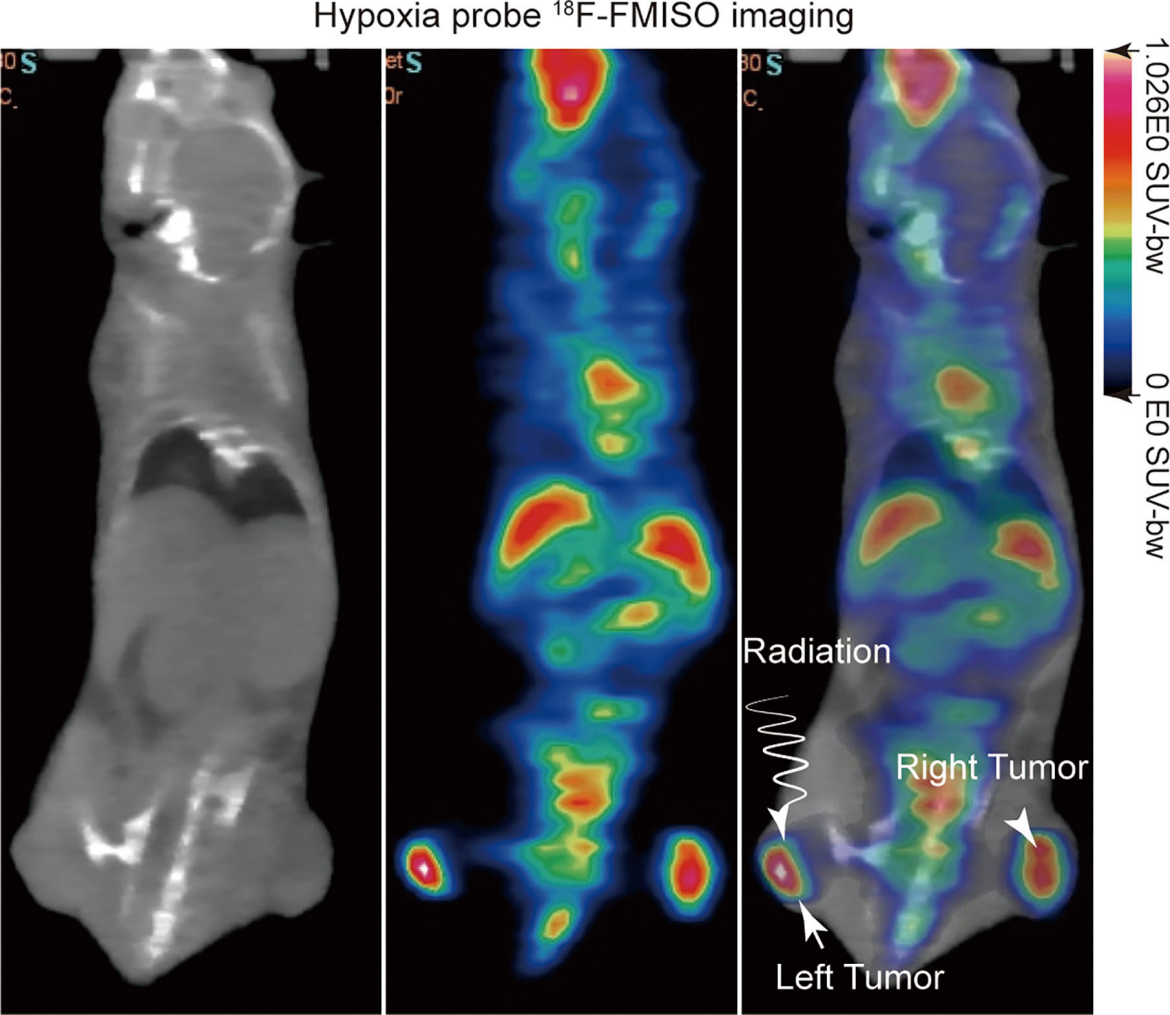
^18^F-FMISO PET/computerized tomography (CT) imaging was performed before irradiation treatment for evaluating the hypoxia status of tumors.

^18^F-FMISO was produced using a modified Explora FDG4 module (Siemens) at SPHIC. For evaluation of the hypoxia status of tumors, micro-PET/CT (Inveon Siemens) scanning was performed on the day before irradiation treatment with an injection of 5.55 MBq (150 μCi) of ^18^F-FMISO through the tail vein (**Figure 1**). ^18^F-FMISO was injected 4 h before the scan (36). Isoflurane was utilized 10 minutes before the scan, and mice were kept under anesthesia during the period of scan. The images were reconstructed with a three dimensional ordered-subset expectation maximization (OSEM3D)/maximum algorithm. The region of interest (ROI) was manually delineated to cover the entire tumor on fused images for data analysis. A similar circular ROI was drawn on the muscle of the opposite hind leg of the mouse on fused images. In order to evaluate the uptake of ^18^F-FMISO in tumors, the maximum standardized uptake value (SUVmax) was calculated by measuring the maximal concentration of radioactivity in ROI. The tumor tissue SUVmax (T), the contralateral normal muscle SUVmax (N), and the ratio of the two values (T/N) were calculated.

### Treatment Planning and Delivery of Carbon Ion Radiotherapy

Mice were assigned to three groups based on randomized experimental designs (37): group I: control group with no treatment; group II: CIRT with open field (CI-OFR group) targeting the whole tumor; group III: partial volume carbon ion microporous radiation (CI-MPR group) targeting hypoxia region. Each group had eight mice. The tumors on left hind legs of mice were irradiated using partial volume CI-MPR or CI-OFT using 20 Gy (physical dose) in a single fraction.

**Figure 2:**
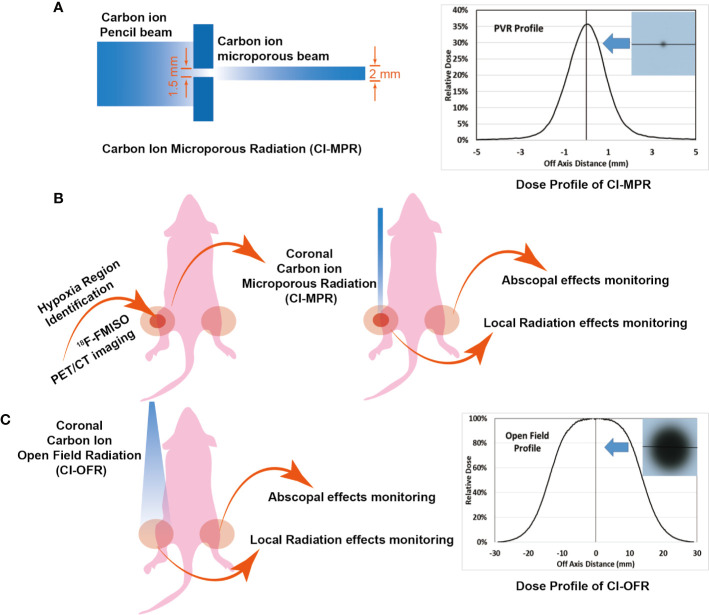
Mouse irradiation and dose profile. **(A, B)** The radiation area and dose profile of carbon ion microporous radiation (CI-MPR) group. **(C)** The radiation area and dose profile of carbon ion open field radiation (CI-OFR) group.

Mice were anesthetized and immobilized on poly(methyl methacrylate) (PMMA) plates. An EBT3 film was attached on plates, opposite to mice side, so that the mice positioning could be monitored by checking the film response after irradiation (**Supplementary Figure 1A**). Then the mouse was placed on a box chamber, where two hind legs of the mouse were perpendicular to the beam axis. To secure precise irradiation of the tumor hypoxia area, we developed a microporous radiation system using a block with a pore in the center (**Supplementary Figure 1B**). The rectangular block 1 with a hole in the center made of aluminum was manufactured for the CI-MPR group. The dimensions of the block were 70.0 mm * 70.0 mm * 20.0 mm, and diameter of hole was 1.5 mm in the center (**Supplementary Figure 1B**). Tuned carbon-ion beams penetrate the target with a relative dose of 35%. The dose profile presented in **Figure 2A** shows that the off-axis distance of the point radiation was 2 mm (full width at half maximum, FWHM). We named this technique carbon ion microporous radiation (CI-MPR). Another block 2 with a gap for the open-field irradiation group, was positioned as close as possible to the mouse. The dimension of the block 2 for open-field radiation group was 120.0 mm * 80.0 mm * 20.0 mm, and the left gap (40.0 mm * 30.0 mm) was left to expose the irradiation targets (**Supplementary Figure 1C**). The dose profile of the open field carbon-ion radiation (CI-OFR) used in the study was shown in **Figure 2C**, and the off-axis distance was 30 mm (FWHM). A horizontal beam was used to protrude through the hole or gap of the block and was adjusted to the isocenter of tumor of the left hind leg by the longitudinal and sagittal laser (see **Supplementary Figures 1B, C**).

CIRT treatment plans were generated in the Syngo (Version 13B, Siemens, Erlangen, Germany) treatment planning system (TPS). The modulated carbon-ion treatment plan had a circular field with a lateral diameter of 20.0 mm. The planned modulation width was 17.0 mm, the planned beam range was 20.0 mm. The prescribed dose to the spread-out Bragg-peak (SOBP) was 20 Gy (physical dose). The output factor of block 1 was calculated with the beam model, and dose profiles of CI-MPR and CI-OFR group were shown in **Figures 2A, C**. Since the plan was designed to treat superficial targets, a PMMA range shifter was used to better the superficial dose distribution. The energies of the carbon beams used were from 116.48 to 141.7 MeV/u, with the corresponding dose averaged LET between 350.07 and 368.82 KeV/μm within the targets.

### Statistical Analysis

The fold change differences of tumor volume on irradiated and unirradiated tumors during the period of observation and on day 15 between the control group and the other two groups were analyzed by two-way ANOVA and two-tailed unpaired Student’s t test, respectively. P values of <0.05 were considered statistically significant.

## Results

### ^18^F-FMISO Micro-PET/Computerized Tomography Imaging

To visualize the tumor hypoxia area, the positron emitted probe ^18^F-FMISO was injected intravenously into a mouse *via* tail vein with a dose of 150 μCi. ^18^F-FMISO PET/CT imaging was performed on tumor-bearing mice before radiation treatment. Typical images are shown in **Figure 1**. The probes were mainly distributed in the center of the tumor, which depicts the hypoxic area of the tumor clearly in the left and right hind leg of mice, with a T/N value of 1.4.

### Carbon-Ion Microporous Radiation

To secure precise irradiation of the tumor hypoxia area, we developed a microporous radiation system called carbon ion microporous radiation (CI-MPR) using a block with a pore in the center with a diameter of 1.5 mm (**Supplementary Figure 1B**). To further protect the area from unnecessary irradiation, we maximized the block size to cover entire body of mouse except for the CIRT field (**Supplementary Figures 1B, C**).

**Figure 3:**
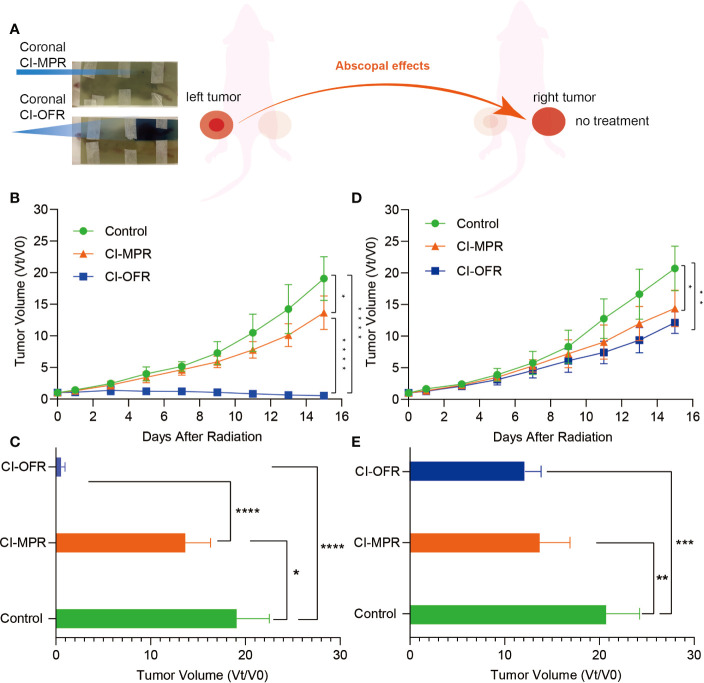
The response after irradiation on EBT3 film and evaluation of tumor volume change of the irradiated and unirradiated tumors. **(A)** The response and one vertex of irradiation could be noted in carbon ion open field radiation (CI-OFR) and carbon ion microporous radiation (CI-MPR) group on the EBT3 film, respectively. **(B)** Tumor volume change on left side (irradiated) during the observation, and p <0.05 and 0.0001 are indicated by * and ****. **(C)** Tumor volume change on right side (unirradiated) during observation, and p <0.05 and 0.01 are indicated by * and **. **(D)** Quantitative analysis of irradiated tumor (left side) volume change on day 15. **(E)** Quantitative analysis of unirradiated tumor (right side) volume change on day 15. Each bar represents the standard error, and p <0.05, 0.01, 0.001, 0.0001 are indicated by *, **, ***, ****.

Moreover, to further maintain radiation accuracy, a piece of EBT3 film was attached on plates, opposite to where the mouse is located, so that the positioning of the mice could be verified by the film response after irradiation. **Figure 3A** showed that the reaction on EBT3 film occurred only in the irradiated area in a case of CI-OFR and CI-MPR group. The outline of irradiated tumor and the left leg could be clearly seen in the CI-OFR group, while in the CI-MPR group, one vertex after irradiation on the EBT3 film, indicating that our radiation technology is feasible and reliable for both CI-MPR and CI-OFR.

### *In Vivo* Abscopal Effects After Carbon Ion Microporous Radiation and Carbon Ion Open Field Radiation

To compare the antitumor immune response to both CI-MPR and CI-OFR for primary and distant tumors, we first established an animal tumor model on both hind legs. When the tumor grew to approximately 7mm x 7mm, the tumor on the left hind leg was irradiated by CI-MPR or CI-OFR. The process of CIRT was shown in **Figure 2**.

The growth of the irradiated tumor (on the left hind leg) and the unirradiated tumor (on the right hind leg), as well as the fold changes of the tumor volumes during the observation and on day 15, were shown in **Figure 3**.

Tumors on both legs of the mice in the control group showed rapid growth as compared to the mice treated with CI-MPR or CI-OFR. Mice treated with CI-MPR or CI-OFR showed that significant growth delay on both the left side (irradiated) and right side (unirradiated) of tumor as compared to the control groups, indicating the direct and abscopal anti-tumor effects of carbon ion beams. Tumor regression of left-sided tumor irradiated with CI-OFR was more prominent as compared to the tumor treated with CI-MPR, and the fold change of the tumor volumes on day 15 in CI-MPR was 25.3 times that of CI-OFR group (P<0.05), demonstrating CI-OFR provided higher local radiation effects than CI-MPR. However, regression of the unirradiated tumor on the right side in both CI-MPR and CI-OFR group was similar, and the fold change of the tumor volumes on day 15 in CI-MPR was 1.1 times that of CI-OFR group (P>0.05), indicating that CI-MPR provided similar abscopal effects as CI-OFR. This phenomenon demonstrated that microporous CIRT with a diameter of 1.5 mm targeting the hypoxic area could achieve similar abscopal effects as open field irradiation.

## Discussion

This present study evaluated the anti-tumor effects triggered by carbon ion microporous radiation targeting hypoxic area with a single high dose on murine tumor model as compared to the conventional open-field tumor technique. The hypoxic area was visualized by ^18^F-FMISO Micro-PET/CT imaging, and the feasibility of the microporous irradiation technique was verified by the EBT3 film and an *in vivo* tumor model. Our study revealed two major new observations: CIRT could induce prominent abscopal effects *in vivo*, and more interestingly, CIRT using our microporous radiation technology could induce abscopal effects similar to CIRT open-field.

To the best of our knowledge, we presented the first *in vivo* evidence of anti-tumor effect of carbon ion microporous irradiation targeting tumor hypoxic area and explored its potential significance, with similar results reported in photon (17, 18, 38). Tubin *et al*. demonstrated that irradiation for hypoxic tumor cells induced higher RIAE compared to the normoxic cells in their preclinical research. Tubin *et al*. and Massaccesi *et al*. also adopted a radiation technique targeting the hypoxic segment of the tumor exclusively with single high dose as palliative treatment in oligometastatic cases or patients with previously irradiated recurrent bulky solid lesions. The researchers observed regression of the irradiated lesions as well as metastatic lesions (unirradiated) after treatment, thereby proving the anti-tumor effect and its validity through this strategy. The authors speculated that targeting the hypoxic region with ionizing radiation may release certain abscopal signals to activate the immune system in comparison with the normoxic region, thus triggering regression of irradiated and unirradiated tumor. As carbon ion beams possess higher RBE and induce more complex DNA damage (70% in the form of double bond break) as compared to photon (23), results of preclinical study have shown that carbon ion beams are more effective than photon beams in eradicating hypoxic tumor cells (28). Our study clearly demonstrated clear growth delays of irradiated and unirradiated tumor after CI-MRP targeting the hypoxic area, and we postulate that CIRT targeting hypoxic region of a tumor may lead to RIAEs in a different manner compared to the photon. However, the differences in the magnitude and mechanism between CIRT and photon beam radiation are awaiting to be investigated.

In the clinical setting, the prescribed dose of the conventional open-field RT for bulky tumors is frequently limited by the surrounding organs at risk. Partial volume tumor irradiation, such as GRID or Lattice, has been shown to evoke anti-tumor immune response and is constantly being applied for improving the therapeutic effect as well as side effects (13, 15, 39). In a clinical investigation, Tubin *et al*. compared the therapeutic effect of a stereotactic body radiotherapy group targeting partial tumor hypoxic segment (SBRT-PATHY) and a conventional palliative radiation group targeting the entire volume tumor for the unresectable stage IIIB/IV bulky non-small cell lung cancer (NSCLC) to similar doses. Interestingly, the control of both the irradiated bulky tumor and distant tumor (unirradiated) were significantly improved in the SBRT-PATHY groups, as compared to those in the palliatively irradiated group (P<0.05) (20). In our study, we observed that the response of the partially irradiated tumor targeting hypoxia in the CI-MPR group was not as significant as the open-field group. The underlying reason of the different observation described by Tublin *et al*. may be a result of, at least in part, the limited irradiated volume in our study which did not cover the entire hypoxic region of the tumor. Additionally, sufficient signaling mediating bystander effect to the unirradiated normoxic region could not be established (13). However, the similar abscopal effects in both the microporous and open-field group demonstrated in our study indicated that carbon ion targeting the hypoxic region, despite its small volume, is sufficient to generate RIAE in the same magnitude. As hypoxic areas are usually located near the center of the tumors, precision radiation therapy using particle beams targeting hypoxic area, even at a very high dose, usually does not result in unnecessary irradiation to the adjacent OARs, thereby substantially improving its therapeutic ratio.

As this is the first reported study on the partial volume particle beam radiation targeting hypoxic region using carbon ion beam, the observations of our study need to be investigated in other tumor models for its generality and specificity. Additionally, investigations that explore the differences in the magnitude between carbon-ion, proton, and photon beams are necessary. At the Shanghai Proton and Heavy Ion Center (SPHIC), studies using various ionizing beam types to explore the local and abscopal effects of hypoxia-targeting partial volume microporous radiation have been initiated using mouse models of glioma, lung cancer, and sarcoma.

Previous studies about RIAEs, induced by partial volume radiation with photon, demonstrated that partial volume irradiation may retain tumor infiltrating lymphocytes in unirradiated areas of the tumor, which can promote stronger anti-tumor responses (40). Additionally, it is suggested that the abscopal effect of partial volume radiation could be mediated by the immunogenic cell death (ICD) and immunogenic modulation (IM) (14). Immunogenic modulation (IM) triggered by radiation contains the upregulation of release of tumor associated antigen (TAAs), the expression of major histocompatibility complex I molecules (MHC-I), the activation of T cells effectively, and the secretion of chemokine and cytokine, thereby altering the tumor microenvironment (TME) and enhancing the anti-tumor immune system function (41). With ongoing investigations at SPHIC, we expect to further illustrate the mechanisms through which partial volume microporous irradiation with carbon ion exerts its immune modulation effect.

Our study demonstrated that biological-guided CIRT using the newly developed microporous technology, targeting tumor hypoxia region only without encompassing the entire tumor volume, could induce robust abscopal effects similar to CIRT covering the entire tumor. The underlying mechanism requires further investigations using animal modes before translating to clinical application.

## Data Availability Statement

The raw data supporting the conclusions of this article will be made available by the authors, without undue reservation.

## Ethics Statement

The animal study was reviewed and approved by Shanghai Proton and Heavy Ion Center Institutional Animal Care and Use Committee.

## Author Contributions

JL, LK, XW, QH, and YS conceived the study and thoroughly revised the manuscript. QH, WW, LL, YH, and JY acquired and analyzed the data. QH, YS, and JL wrote the manuscript. All authors contributed to the article and approved the submitted version.

## Funding

This work was mainly supported by the National Key Research and Development Program of China (no. 2018YFC0115700), the Science and Technology Commission of Shanghai Municipality (no.18XD1423000), the Program of Shanghai Technology Research Leader (no.19XD1432900) and the Natural Science Foundation Project of Shanghai Science and Technology Innovation Action Plan (no.20ZR1453400). All funding sources should be provided, including grant numbers if applicable. Please ensure to add all necessary funding information, as after publication this is no longer possible.

## Conflict of Interest

The authors declare that the research was conducted in the absence of any commercial or financial relationships that could be construed as a potential conflict of interest.

## Supplementary Material

Supplementary Figure 1 Mouse positioning and irradiation device. **(A)** Mice were anesthetized and immobilized on PMMA plates. An EBT3 film piece was attached on plates, opposite to mice side, so that the mice positioning could be monitored by checking the film response after irradiation. **(B)** The dimension of the block 1 for CI-MPR group. **(C)** The dimension of the block 2 for CI-OFR group, and the left gap was left to expose the irradiation targets.
