# Supplementary figures and images for: Biological Guided Carbon-Ion Microporous Radiation to Tumor Hypoxia Area Triggers Robust Abscopal Effects as Open Field Radiation
Source: Front Oncol. 2020 Nov 19;10:597702. doi: 10.3389/fonc.2020.597702 (PMC7713593; doi:10.3389/fonc.2020.597702)

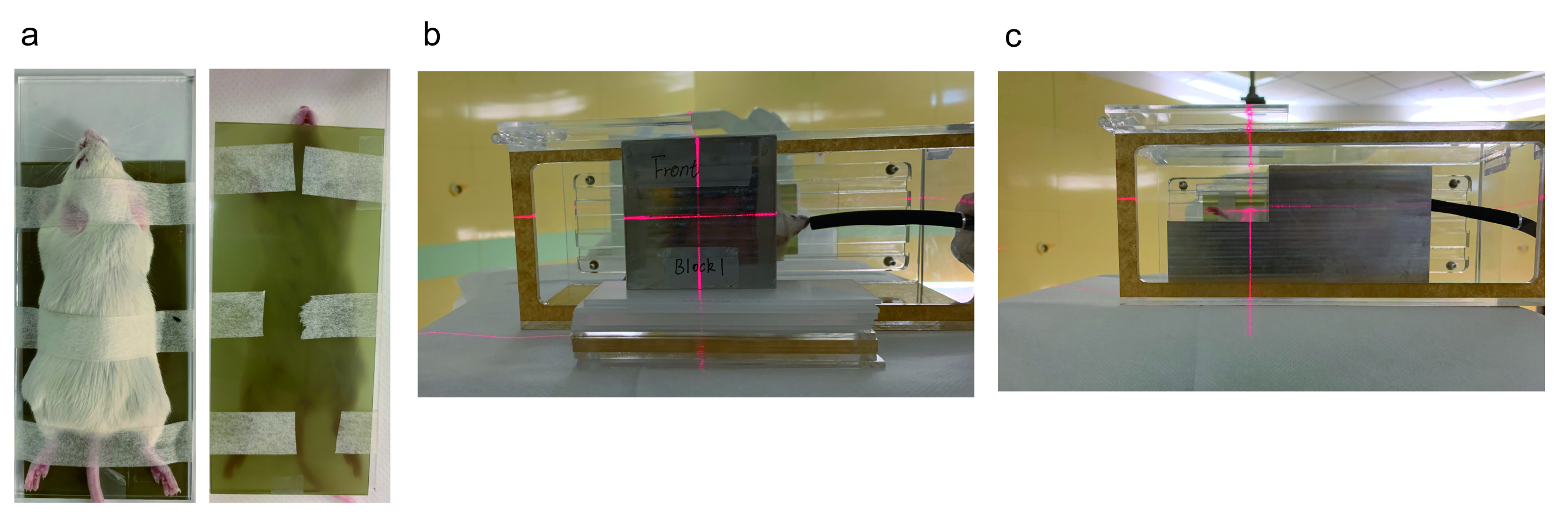

Supplement: Supplementary Figure 1 — Mouse positioning and irradiation device. (A) Mice were anesthetized and immobilized on PMMA plates. An EBT3 film piece was attached on plates, opposite to mice side, so that the mice positioning could be monitored by checking the film response after irradiation. (B) The dimension of the block 1 for CI-MPR group. (C) The dimension of the block 2 for CI-OFR group, and the left gap was left to expose the irradiation targets. [file Image_1.tif]
